# Association of MTHFR C677T and A1298C Polymorphisms with Glaucoma Risk: a Systematic Review Meta-Analysis based 42 Case-Control Studies

**Published:** 2019

**Authors:** Mohsen Gohari, Seyed Alireza Mirjalili, Mohammad Javad Akbarian-Bafghi, Mohammad Hossein Jarahzadeh, Masoud Zare-Shehneh, Hossein Neamatzadeh

**Affiliations:** * Geriatric Ophthalmology Research Center, Shahid Sadoughi University of Medical Sciences, Yazd, Iran; ** Department of Medical Genetics, Shahid Sadoughi University of Medical Sciences, Yazd, Iran; *** Department of Health Care Management, Bam University of Medical Sciences, Bam, Iran; **** Department of Anesthesiology and Critical Care, Shahid Sadoughi University of Medical Sciences, Yazd, Iran

**Keywords:** glaucoma, methylenetetrahydrofolate reductase, polymorphism, meta-analysis

## Abstract

**Aim:** Several epidemiological studies have been performed to explore the association of MTHFR polymorphisms with glaucoma risk. However, the results were inconsistent or even inconclusive. Hence, we performed a meta-analysis to evaluate the association of MTHFR C677T and A1298C polymorphisms with glaucoma risk.

**Methods:** A comprehensive literature search on PubMed, Google Scholar, EMBASE, and CNKI databases was performed to find all eligible studies up to January 30, 2019. The pooled odds ratios (ORs) with 95% confidence intervals (CIs) were used to assess the strength of such association.

**Results:** A total of 42 case-control studies including 33 studies for MTHFR C677T and nine studies for A1298C polymorphism were selected. Pooled results showed that there was no significant association between the MTHFR C677T polymorphism and glaucoma risk. Similarly, no associations were found in subgroup analysis based on ethnicity and glaucoma type. However, there was a significant association between the A1298C polymorphism and the increased risk of glaucoma under heterozygote model (OR=0.765, 95% CI=0.626-0.935, P=0.009). Moreover, the significant association between MTHFR A1298C polymorphism and glaucoma were found by ethnicity and primary open angle glaucoma (POAG).

**Conclusions:** The present meta-analysis revealed that MTHFR A1298C polymorphism is significantly associated with the increased risk of glaucoma, but not MTHFR C677T polymorphism.

## Introduction

Glaucoma is an optic neuropathy in which the optic nerve is damaged with typical loss of nerve fibers and increasing cupping of the optic disc, leading to progressive, irreversible loss of vision [**1**,**2**]. A leading cause of all blindness worldwide, secondary to cataracts, glaucoma is the main cause of irreversible vision loss [**3**]. It is estimated that more than 60 million people had glaucoma in 2010, 8.4 million of whom are bilaterally blind as a result of this disease [**4**]. In general, glaucoma might be classified in three major categories: primary open angle glaucoma (POAG), primary congenital glaucoma (PCG) and primary angle-closure glaucoma (PACG) [**5**].

Glaucoma is a multifactorial disease involving both environmental and genetic factors [**6**,**7**]. During the past decade, molecular genetic studies of glaucoma have yielded some success. The importance of genetic factors in the etiology of glaucoma is supported by genome-wide association studies (GWASs) [**8**]. Recently, several candidate novel loci have been identified in a GWAS for POAG (e.g., ABCA1, AFAP1, GMDS, PMM2, TGFBR3, FNDC3B, ARHGEF12, GAS7, FOXC1, ATXN2, TXNRD2); PACG (e.g., EPDR1, CHAT, GLIS3, FERMT2, DPM2-FAM102); and exfoliation syndrome (XFS) glaucoma (CACNA1A) [**8**,**9**]. Furthermore, several epidemiological studies have reported a link between methylenetetrahydrofolate reductase (MTHFR) gene polymorphisms and glaucoma [**10**,**11**].

The MTHFR gene is located on chromosome 1p36.3 [**12**,**13**]. It is an important regulatory enzyme in the folate related one carbon metabolism, which is responsible for catalyzing 5, 10-methylenetetrahydrofolate to 5-methyltetrahydrofolate [**14**,**15**]. In addition, MTHFR plays an important role by directing folate metabolites through the DNA methylation pathways [**12**]. An increased level of plasma homocysteine (Hcy) has been observed in patients with glaucoma [**16**]. The MTHFR gene is encoded by 11 exons and includes several SNP, some of which have functional relevance and result in high Hcy level. Many studies have shown an increased risk of glaucoma in patients with MTHFR C677T and A1298C polymorphism. However, results from these studies were inconsistent or inconclusive. It was suggested that this inconsistency might be related to the single studies with low statistical power, publication biases, and ethnicity differences. Thus, we have performed the current systematic review and meta-analysis to collecting and summarizing the evidence on the association of MTHFR C677T and A1298C polymorphisms with the risk of glaucoma.

## Materials and Methods

**Study Identification and Selection**

We have performed a comprehensive literature search using PubMed, Web of Science, Google Scholar, Cochrane Library, Embase, and Chinese Biomedical Literature database (CBM) databases to identify studies that evaluated the association between MTHFR C677T polymorphism and the risk of glaucoma up to October 2018, with the following keywords: “Methylenetetrahydrofolate reductase”, “MTHFR”, “MTHFR C677T”, or “MTHFR A1298C” and “polymorphism”, “mutation”, or “variant” and “glaucoma” and “primary open-angle glaucoma” or “POAG” and “pseudoexfoliation glaucoma” or “PXFG”, “pseudoexfoliation syndrome with glaucoma” or “PEXG” and “normal-tension glaucoma” or “NTG” and “primary angle-closure glaucoma” or “PACG” and “primary angle-closure glaucoma” or “PACG”, “high-tension glaucoma” or “HTG”, and “juvenile-onset open-angle glaucoma” or “JOAG”. We have retrieved any article matching the keywords and we evaluated it by reading the title and abstract. In addition, we have screened the references lists of the retrieved articles for original papers.

**Inclusion and Exclusion Criteria**

The following criteria were used for the study selection: 1) a case-control study evaluating the association of MTHFR C677T and A1298C polymorphisms with the risk of glaucoma and its types; 2) case-control or cohort studies; 3) sufficient data for estimating an odds ratio (OR) with 95% confidence interval (CI); 4) no overlapping data. In addition, if studies had the same or overlapping data, we have included only the largest study in the final analysis. The major excluding criteria for studies were the following: (1) not glaucoma research, (2) reviews, letters or case reports, (3) duplicate of previous publication, and (4) and those articles without definite information of genotypes.

**Data Extraction**

We have extracted information carefully from all the eligible studies independently by two investigators based on the above listed inclusion criteria. The following data were collected from each study: the first author’s name, the year of publication, ethnicity, country of origin, glaucoma type, genotyping method, source of control groups (population-based or hospital-based controls), total number of cases and controls, the frequencies of genotypes, minor allele frequencies (MAFs), and Hardy-Weinberg equilibrium (HWE) test in control subjects. Allele frequencies were calculated from the corresponding genotype distributions using an online website. Finally, the extracted data in terms of accuracy and any discrepancy between these two authors was resolved by reaching a consensus through discussion or the involvement of a third author who made the final decision through discussions. 

**Statistical Analysis**

Pooled odds ratios (ORs) and corresponding 95% confidence intervals (CIs) were calculated to assess the association of MTHFR C677T and A1298C polymorphisms with the risk of glaucoma. The significance of the pooled OR was determined by the Z-test. The pooled ORs were performed under five genetic models, i.e., allele (B vs. A), homozygote (BB vs. AA), heterozygote (BA vs. AA), dominant (BB+BA vs. AA), and recessive (BB vs. BA+AA), which a “A” denotes a major allele; “B” denotes a minor allele. Heterogeneity (between-study inconsistency) was assessed by the Cochran Χ2-based Q test (Heterogeneity was considered statistically significant if P<0.10) and the I2 statistics. An I2 value of 0% represents no heterogeneity, with values of 25%, 50%, 75%, or more represent low, moderate, high, and ex¬treme heterogeneity, respectively. A fixed effect model (Mantel-Haenszel method) was used to calculate pooled OR when there was no heterogeneity among the studies. Otherwise, the fixed-effects model (Mantel-Haenszel approach) was used. We have calculated the Hardy-Weinberg equilibriums (HWEs) with goodness-of-fit tests (i.e., chi-square or Fisher’s exact tests). In addition, one-way sensitivity analyses were carried out by consecutively omitting one study at a time to assess power of the meta-analysis [**15**]. In addition, sensitivity analysis was also performed, excluding studies whose allele frequencies in controls exhibited a significant deviation from the Hardy–Weinberg equilibrium (HWE), given that the deviation may denote bias. Deviation of HWE may reflect methodological problems such as genotyping errors, population stratification or selection bias. Visual inspection of the asymmetry of funnel plots was carried out to assess potential publication bias. Begg’s funnel plot, a scatter plot of effect against a measure of study size was used as a visual aid to detect bias or systematic heterogeneity. Publication bias was assessed by Egger’s test (p<0.05 was considered statistically significant). If publication bias existed, the Duval and Tweedie non-parametric “trim and fill” method was used to adjust for it. A meta-regression analysis was carried out to identify the major sources of between-studies variation in the results, using the log of the ORs from each study as dependent variables, and ethnicity and source of controls as the possible sources of heterogeneity. All the statistical calculations were performed using Comprehensive Meta-Analysis (CMA) software version 2.0 (Biostat, USA). Two-sided P-values < 0.05 were considered statistically significant.

## Results

**Study Selection and Characteristics**

**Fig. 1:**
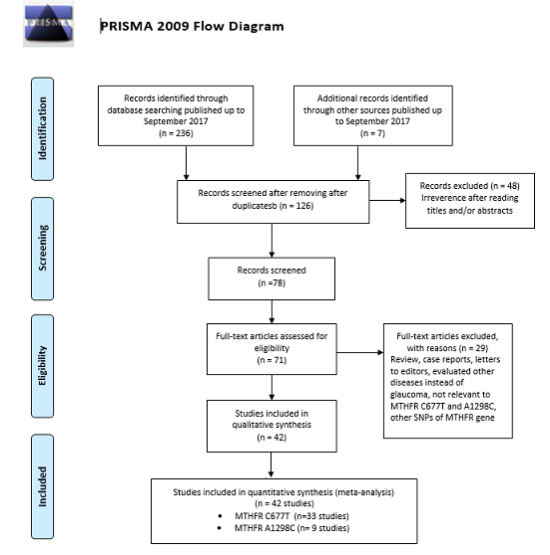
Flow chart of literature search and study selection

A flow diagram schematizing the inclusion and exclusion process of identified articles with the inclusion criteria is presented in **Fig. 1**

After a comprehensive search, a total of 342 articles were identified. Of these studies, the first screening excluded 216 as duplicates or not relevant, leaving 126 for further selection. Among the remaining studies, 84 articles were excluded because they were review articles, letters to editors, previous meta-analyses, not relevant to MTHFR C677T and A1298C, not case-control studies, evaluated other diseases instead of glaucoma, case reports, and other polymorphisms of MTHFR gene. Finally, a total of 42 case-control studies including 33 studies (in 19 publications) with 3,504 cases and 2,525 controls for MTHFR C677T [**9**–**11**,**17**–**31**] and nine studies (in six publications) with 1,073 cases and 775 controls for A1298C [**11**,**19**–**21**,**23**,**29**] were selected. The main characteristics of studies included in the current meta-analysis are presented in **Tables 1** and **2**.

**Table 1:** Characteristics of the studies included in the MTHFR C677T polymorphism meta-analysis

First author	Country (Ethnicity)	Type	Case	Control	SOC	Genotyping Technique			Cases					Control			MAFs	HWE
							Genotypes			Alleles		Genotypes			Alleles			
							CC	TC	TT	C	T	CC	TC	TT	C	T		
Bleich 2002	Germany (Caucasian)	POAG	18	19	PB	RT–PCR	5	11	2	21	15	13	5	1	31	7	0.184	0.587
Jünemann 2005	Germany (Caucasian)	POAG	76	71	HB	RT–PCR	32	37	7	101	51	45	24	2	114	28	0.197	0.568
		PEXG	71				36	29	6	101	41							
Mossbock 2006	Austria (Caucasian)	POAG	204	211	HB	PCR-RFLP	119	71	14	309	99	105	86	20	296	126	0.298	0.695
		PXFG	138				72	50	16	194	82							
Mabuchi 2006	Japan (Asian)	POAG	133	106	HB	Sequencing	51	55	27	157	109	48	39	19	135	77	0.363	0.035
		NTG	131				54	58	19	166	96							
Fingert 2006	USA (Caucasian)	POAG	178	166	PB	PCR-RFLP	72	77	29	221	135	75	73	18	223	109	0.328	0.969
		PEXG	45				12	29	4	53	37							
Zetterberg 2007	Estonia (Caucasian)	POAG	243	187	HB	Sequencing	126	97	20	349	137	89	75	23	253	121	0.323	0.252
Fan 2008	USA (Caucasian)	PXFG	61	50	HB	TaqMan	23	31	7	78	44	21	22	7	64	36	0.360	0.749
Michael 2008	Pakistan (Asian)	POAG	90	70	HB	PCR-RFLP	70	20	0	160	20	57	13	0	127	13	0.092	0.391
		PACG	60				48	8	4	104	16							
Michael 2009	Pakistan (Asian)	POAG	173	143	HB	PCR-RFLP	123	49	1	295	51	101	41	1	243	43	0.150	0.143
		PACG	122				84	26	12	194	50							
Clement 2009	Australia (Caucasian)	POAG	36	42	PB	RT–PCR	17	14	5	48	24	25	14	3	64	20	0.238	0.598
		PXFG	48				18	23	7	59	37							
		NTG	34				21	11	2	53	15							
Woo 2009	Korea (Asian)	NTG	78	100	HB	PCR-RFLP	25	34	19	84	72	31	50	19	112	88	0.440	0.883
Fan 2010	Hong Kong (Asian)	HTG	255	201	PB	Sequencing	11	87	154	110	400	6	60	135	72	330	0.820	0.829
		NTG	100				5	30	64	40	160							
		JOAG	50				0	20	26	22	78							
Nilforoushan 2012	Iran (Asian)	POAG	73	90	HB	Sequencing	39	28	6	106	40	53	33	4	139	41	0.227	0.688
		PXFG	85				46	31	8	123	47							
Shi 2013	China (Asian)	PACG	231	306	HB	TaqMan	81	106	44	268	194	93	152	61	338	274	0.447	0.937
Buentello 2013	Mexico (Latinos)	POAG	118	100	HB	Sequencing	23	53	42	99	137	17	49	34	83	117	0.585	0.926
Gupta 2014	India (Asian)	POAG	144	173	HB	PCR-RFLP	101	35	8	237	51	137	34	2	308	38	0.109	0.946
		PACG	87				73	14	0	160	14							
Zacharaki 2014	Greece (Caucasian)	POAG	64	130	HB	TaqMan	22	31	11	75	53	39	70	21	148	112	0.430	0.263
		PXFG	72				29	33	10	91	53							
Al-Shahrani 2015	Saudi Arabia (Asian)	POAG	144	280	NR	PCR-RFLP	88	56	0	232	56	210	70	0	490	70	0.125	0.016
		PACG	66				49	17	0	115	17							
Dixit 2015	India (Asian)	POAG	80	80	HB	PCR-RFLP	49	30	1	128	32	30	48	2	108	52	0.325	0.001
*POAG = primary open angle glaucoma, PXFG = pseudoexfoliation glaucoma, PEXG = pseudoexfoliation syndrome with glaucoma, NTG = normal-tension glaucoma, PACG = primary angle-closure glaucoma, HTG = high-tension glaucoma, JOAG = juvenile-onset open-angle glaucoma, SOC = source of control, PCR–RFLP = Polymerase chain reaction-restriction fragment length polymorphism, RT–PCR = Real time-polymerase chain reaction, NR = Not report, PB = Population–based, HB = Hospital–based, MAFs = Minor Allele Frequency, HWE = Hardy–Weinberg equilibrium in control population*																		

**Table 2:** Characteristics of the studies included in the MTHFR A1298C polymorphism meta-analysis

First author	Country (Ethnicity)	Type	Case	Control	SOC	Genotyping Technique			Cases					Control			MAFs	HWE
							Genotypes			Alleles		Genotypes			Alleles			
							AA	AC	CC	A	C	AA	AC	CC	A	C		
Mabuchi 2006	Japan(Asian)	POAG	133	106	HB	Sequencing	87	43	3	217	49	61	44	1	166	46	0.217	0.022
		NTG	131				80	51	0	211	51							
Zetterberg 2007	Estonia(Caucasian)	POAG	243	187	HB	Sequencing	119	97	27	335	151	88	87	12	263	111	0.296	0.117
Fan 2008	USA (Caucasian)	PXFG	57	50	HB	TaqMan	26	20	11	72		22	19	9	63	37	0.370	0.191
Micheal 2009	Pakistan(Asian)	POAG	173	146	HB	PCR-RFLP	35	114	24	184	162	20	97	26	140	152	0.521	≤0.001
		PACG	122				34	76	12	144	100							
Woo 2009	Korea(Asian)	NTG	78	156	HB	PCR-RFLP	57	19	2	133	23	75	22	3	172	28	0.140	0.387
Zacharaki 2014	Greece(Caucasian)	POAG	64	130	HB	TaqMan	11	31	22	53	75	21	70	39	112	148	0.569	0.263
		PXFG	72				10	33	29	53	91							
*POAG = primary open angle glaucoma, PXFG = pseudoexfoliation glaucoma, NTG = normal-tension glaucoma, PACG = primary angle-closure glaucoma, SOC = source of control, PCR–RFLP = Polymerase chain reaction-restriction fragment length polymorphism, HB = Hospital–based, MAFs = Minor Allele Frequency, HWE = Hardy–Weinberg equilibrium in control population*																		

Among these studies, six types of glaucoma, including primary open angle glaucoma (POAG), pseudoexfoliation glaucoma (PXFG) or pseudoexfoliation syndrome with glaucoma (PEXG), normal-tension glaucoma (NTG), primary angle-closure glaucoma (PACG), high-tension glaucoma (HTG), and juvenile-onset open-angle glaucoma (JOAG) were involved. Among the selected studies, 23 case-control studies were conducted in the Asians, 18 studies were conducted in the Caucasians, and one study was conducted in the Latinos. Genotyping methods used in the studies include PCR-RFLP, Real-time PCR, TaqMan, and sequencing. The genotype frequencies in the control group for three publications did not fit well in the Hardy-Weinberg equilibrium (P>0.05).

**Quantitative Synthesis**

**MTHFR C677T Polymorphism**

**Fig. 2:**
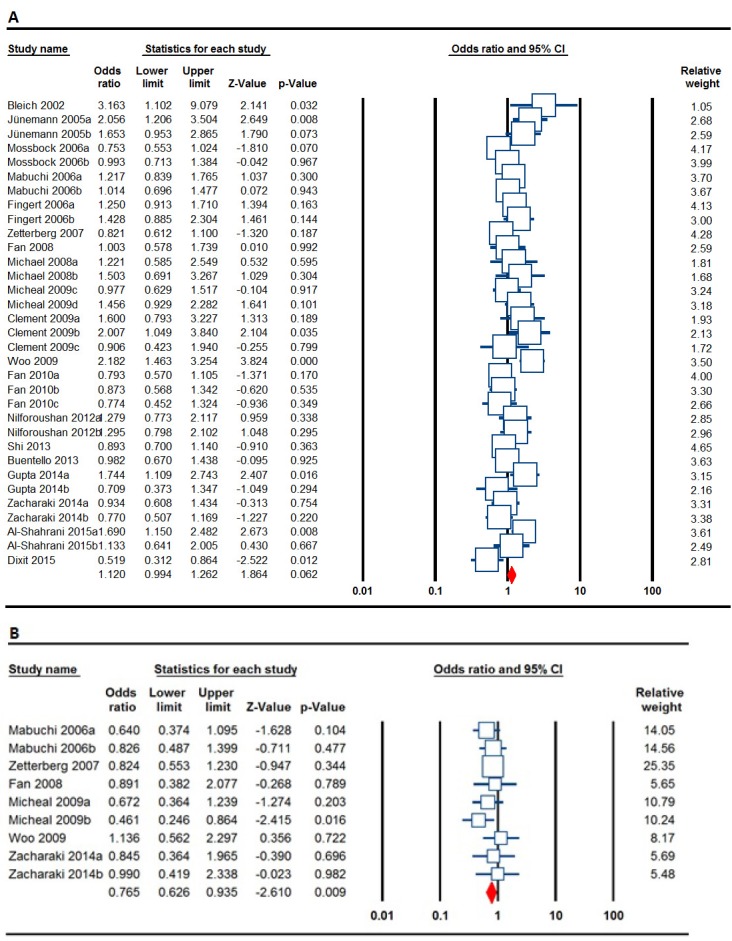
Forest plots for the association of MTHFR C677T and A1298C polymorphisms with risk of risk glaucoma. A: MTHFR C677T (allele model: T vs. C); B: MTHFR A1298C (heterozygote model: CA vs. AA)

**Table 3** listed the main results of the meta-analysis of MTHFR C677T polymorphism and glaucoma risk. After the 33 case-control studies were pooled into meta-analysis, no evidence of a significant association between MTHFR C677T polymorphism and glaucoma risk was observed under all genetic models (T vs. C: OR = 1.120. 95% CI 0.994-1.262, P = 0.062, **Fig. 2A**; TT vs. CC: OR = 1.081. 95% CI 0.899-1.299, P = 0.410; TC vs. CC: OR = 1.033. 95% CI 0.899-1.188, P = 0.646; TT+TC vs. CC: OR = 1.113. 95% CI 0.948-1.306, P = 0.193; TT vs. TC+CC: OR = 1.015. 95% CI 0.876-1.175, P = 0.845).

**Table 3:** Summary risk estimates for association between MTHFR C677T polymorphism and glaucoma risk

Subgroup	Genetic Model	Type of Model	Heterogeneity		Odds ratio				Publication Bias	
			I2 (%)	PH	OR	95% CI	ZOR	POR	PBeggs	PEggers
Overall	T vs. C	Random	56.96	≤0.001	1.120	0.994-1.262	1.864	0.062	0.052	0.031
	TT vs. CC	Fixed	26.28	0.095	1.081	0.899-1.299	0.824	0.410	0.010	0.008
	TC vs. CC	Random	34.76	0.027	1.033	0.899-1.188	0.460	0.646	0.168	0.219
	TT+TC vs. CC	Random	55.75	≤0.001	1.113	0.948-1.306	1.303	0.193	0.752	0.470
	TT vs. TC+CC	Fixed	31.31	0.054	1.015	0.876-1.175	0.195	0.845	0.022	0.005
By Glaucoma Type										
POAG	T vs. C	Random	66.56	≤0.001	1.199	0.983-1.462	1.791	0.073	0.373	0.152
	TT vs. CC	Fixed	36.99	0.087	1.120	0.853-1.470	0.816	0.414	0.200	0.145
	TC vs. CC	Random	59.03	0.002	1.127	0.887-1.431	0.977	0.329	0.322	0.384
	TT+TC vs. CC	Random	64.72	≤0.001	1.149	0.898-1.470	1.105	0.269	0.428	0.264
	TT vs. TC+CC	Fixed	11.19	0.333	1.124	0.878-1.439	0.927	0.354	0.582	0.166
PACG	T vs. C	Fixed	29.71	0.223	0.99	0.828-1.200	-0.033	0.974	0.806	0.501
	TT vs. CC	Random	69.18	0.021	2.356	0.407-13.650	0.956	0.339	1.000	0.359
	TC vs. CC	Fixed	0.00	0.946	0.820	0.636-1.056	-1.537	0.124	0.806	0.971
	TT+TC vs. CC	Fixed	0.00	0.807	0.903	0.710-1.149	-0.828	0.408	1.000	0.458
	TT vs. TC+CC	Random	68.98	0.022	2.594	0.457-14.733	1.076	0.282	1.000	0.363
PXFG + PEXG	T vs. C	Fixed	39.66	0.127	1.151	0.965-1.372	1.563	0.118	0.133	0.080
	TT vs. CC	Fixed	9.68	0.355	1.271	0.843-1.914	1.145	0.252	0.308	0.284
	TC vs. CC	Fixed	45.22	0.090	1.101	0.859-1.411	0.761	0.447	0.734	0.320
	TT+TC vs. CC	Random	52.93	0.047	1.295	0.908-1.847	1.426	0.154	0.734	0.372
	TT vs. TC+CC	Fixed	0.00	0.591	1.208	0.820-1.780	0.956	0.339	0.734	0.249
NTG	T vs. C	Random	74.92	0.008	1.179	0.742-1.871	0.697	0.486	1.000	0.744
	TT vs. CC	Fixed	0.00	0.610	1.019	0.698-1.488	0.096	0.923	0.308	0.202
	TC vs. CC	Fixed	0.00	0.771	0.923	0.567-1.502	-0.323	0.746	0.734	0.503
	TT+TC vs. CC	Random	68.19	0.024	1.217	0.634-2.339	0.590	0.555	0.308	0.512
	TT vs. TC+CC	Fixed	51.54	0.103	1.069	0.756-1.512	0.78	0.706	0.308	0.912
By ethnicity										
Asian	T vs. C	Random	60.66	0.00	1.113	0.941-1.317	1.251	0.211	0.939	0.622
	TT vs. CC	Fixed	31.93	0.113	1.105	0.842-1.450	0.723	0.470	0.198	0.149
	TC vs. CC	Fixed	35.16	0.071	1.006	0.833-1.214	0.059	0.953	0.448	0.598
	TT+TC vs. CC	Random	58.36	0.001	1.063	0.851-1.329	0.539	0.590	0.081	0.151
	TT vs. TC+CC	Random	52.48	0.009	1.146	0.821-1.599	0.798	0.425	0.198	0.053
Caucasian	T vs. C	Random	57.90	0.004	1.139	0.946-1.72	1.373	0.170	0.028	0.007
	TT vs. CC	Fixed	35.06	0.102	1.088	0.829-1.428	0.607	0.544	0.044	0.022
	TC vs. CC	Random	46.01	0.035	1.094	0.8858-1.394	0.723	0.470	0.017	0.010
	TT+TC vs. CC	Random	60.39	0.003	1.195	0.914-1.562	1.300	0.194	0.032	0.010
	TT vs. TC+CC	Fixed	1.848	0.428	1.087	0.837-1.412	0.626	0.531	0.246	0.077

In the subgroup analysis by glaucoma type, no significant associations with POAG, PACG, PXFG, and NTG subgroups were observed. Moreover, no significant association was found in a subgroup analysis by ethnicity among Asian and Caucasian populations (**Table 3**). The studies were further stratified based on genotyping technique, source of control subjects and HWE. In the PCR-RFLP group, significantly increased association between *MTHFR C677T* polymorphism and glaucoma risk were found in the recessive model (TT vs. TC+CC: OR = 1.438. 95% CI 1.056-1.958, P = 0.021). The population based subgroup analysis also revealed that the presence of the *MTHFR C677T*, which was related to a higher risk of glaucoma under the heterozygote model (TT vs. TC: OR = 1.350, 95% CI 1.012-1.802, P = 0.041). Subgroup analysis of studies in accordance with HWE showed that there was a significant association between *MTHFR C677T* polymorphism and the increased risk of glaucoma under the allele model (OR = 1.156, 95% CI 1.020-1.309, p = 0.023) (data not shown).

**MTHFR A1298C Polymorphism**

**Table 4** listed the main results of the meta-analysis of *MTHFR A1298C* polymorphism and glaucoma risk. When all the eligible studies were pooled into the meta-analysis of *MTHFR A1298C* polymorphism, significantly increased risk of glaucoma was observed in the heterozygote model (CA vs. AA: OR = 0.765, 95% CI 0.626-0.935, p = 0.009, **Fig. 2B**). Table 4 also summarizes the results of the subgroup analyses by ethnicity and types of glaucoma. When stratified by ethnicity, a significant association between *MTHFR A1298C* polymorphism and increased risk of glaucoma was detected among Asians (C vs. A: OR = 0.826, 95% CI 0.692-0.987, p = 0.036; CC vs. AA: OR = 0.456, 95% CI 0.268-0.777, p = 0.004; and CA vs. AA: OR = 705, 95% CI 0.541-0.918, p = 0.010) and Caucasians (CC vs. CA+AA: OR = 1.443, 95% CI 1.019-2.044, p = 0.039). In addition, when stratifying by types of glaucoma, we found that *MTHFR A1298C* was significantly associated with POAG risk under heterozygote model (CA vs. AA: OR = 0.746, 95% CI 0.570-0.976, p= 0.033), but not with PXFG and NTG (**Table 4**). Moreover, subgroup analysis of studies in agreement with HWE showed that there was a significant association between *MTHFR A1298C* polymorphism and increased risk of glaucoma under the recessive model (OR = 1.440, 95% CI 1.023-2.026, p = 0.037) (data not shown).

**Table 4:** Summary risk estimates for association between MTHFR A1298C polymorphism and glaucoma risk

Subgroup	Genetic Model	Type of Model	Heterogeneity		Odds ratio				Publication Bias	
			I2 (%)	PH	OR	95% CI	ZOR	POR	PBeggs	PEggers
Overall	C vs. A	Fixed	42.53	0.084	0.943	0.826-1.075	-0.880	0.379	0.602	0.257
	CC vs. AA	Fixed	44.50	0.072	0.878	0.627-1.231	-0.753	0.425	0.602	0.909
	CA vs. AA	Fixed	0.00	0.753	0.765	0.626-0.935	-2.610	0.009	0.602	0.666
	CC+CA vs. AA	Fixed	26.84	0.205	0.873	0.721-1.058	-1.381	0.167	0.602	0.620
	CC vs. CA+AA	Fixed	24.92	0.222	1.083	0.824-1.422	0.571	0.568	0.754	0.924
By Type										
POAG	C vs. A	Fixed	0.00	0.507	0.939	0.787-1.120	-0.700	0.484	0.734	0.857
	CC vs. AA	Fixed	38.10	0.183	1.027	0.655-1.611	0.115	0.908	1.000	0.767
	CA vs. AA	Fixed	0.00	0.861	0.746	0.570-0.976	-2.138	0.033	1.000	0.763
	CC+CA vs. AA	Fixed	0.00	0.837	0.832	0.643-1.077	-1.394	0.163	1.000	0.673
	CC vs. CA+AA	Fixed	26.28	0.254	1.153	0.798-1.666	0.757	0.449	0.308	0.539
PXFG	C vs. A	Fixed	0.00	0.449	1.179	0.844-1.646	0.966	0.334	NA	NA
	CC vs. AA	Fixed	0.00	0.558	1.313	0.665-2.591	0.785	0.432	NA	NA
	CA vs. AA	Fixed	0.00	0.864	0.938	0.513-1.715	-0.217	0.836	NA	NA
	CC+CA vs. AA	Fixed	0.00	0.670	1.050	0.601-1.833	0.170	0.865	NA	NA
	CC vs. CA+AA	Fixed	0.00	0.530	1.422	0.852-2.374	1.346	0.178	NA	NA
NTG	C vs. A	Fixed	70.83	0.064	1.204	0.608-2.382	0.532	0.595	NA	NA
	CC vs. AA	Fixed	0.00	0.512	0.650	0.133-3.171	-0.533	0.594	NA	NA
	CA vs. AA	Fixed	0.00	0.477	0.926	0.607-1.413	-0.356	0.722	NA	NA
	CC+CA vs. AA	Fixed	71.59	0.061	1.178	0.783-1.773	0.785	0.432	NA	NA
	CC vs. CA+AA	Fixed	0.00	0.391	0.910	0.188-4.402	-0.118	0.906	NA	NA
By Ethnicity										
Asians	C vs. A	Fixed	52.89	0.075	0.826	0.692-0.987	-2.099	0.036	0.220	0.115
	CC vs. AA	Fixed	0.00	0.433	0.456	0.268-0.777	-2.888	0.004	0.806	0.473
	CA vs. AA	Fixed	0.420	0.404	0.705	0.541-0.918	-2.592	0.010	1.000	0.750
	CC+CA vs. AA	Random	59.70	0.042	0.829	0.554-1.241	-0.910	0.363	1.000	0.417
	CC vs. CA+AA	Fixed	0.00	0.593	0.686	0.442-1.063	-1.686	0.092	0.806	0.576
Caucasian	C vs. A	Fixed	0.00	0.852	1.106	0.909-1.345	1.004	0.316	0.734	0.997
	CC vs. AA	Fixed	0.00	0.824	1.362	0.881-2.106	1.390	0.165	0.308	0.196
	CA vs. AA	Fixed	0.00	0.985	0.856	0.628-1.167	-0.984	0.325	0.089	0.242
	CC+CA vs. AA	Fixed	0.00	0.956	0.959	0.715-1.286	-0.279	0.780	0.308	0.465
	CC vs. CA+AA	Fixed	0.00	0.783	1.443	1.019-2.044	2.066	0.039	1.000	0.578
*NA = Not Applicable*										

**Minor Allele Frequencies (MAFs)**

The minor allele frequencies (MAFs) of the MTHFR C677T and A1298C polymorphisms by ethnicity are presented in **Tables 1** and **2**. The allele and genotype distributions of MTHFR C677T and A1298C polymorphisms exhibited ethnic variations. The 677T allele frequencies in the Caucasian and Asians populations were 30.7% (18.4%-43.0%) and 22.75% (9.2%-36.3%), respectively. The 1298C allele frequencies in the Caucasian and Asians populations were 34.3% (11.7%-56.9%) and 17.85% (14.0%-21.7%), respectively. Therefore, the frequencies of the 677T and 1298C alleles in Asians were less than in Caucasians.

**Heterogeneity Test and Sensitivity Analyses**

There was a significant heterogeneity among these studies for *MTHFR C677T* polymorphism under allele model comparison (T vs. C: Ph = ≤ 0.001), homozygote model comparison (TT vs. CC: Ph = 0.005) and dominant model comparison (TT + CT vs. CC: Ph = 0.001). Then, we assessed the source of heterogeneity by meta-regression analysis. However, we found that ethnicity, glaucoma types, genotyping methods, source of controls and HWE did not contribute to substantial heterogeneity among the meta-analysis (**Table 2**). Sensitivity analyses were conducted to determine whether modification of the inclusion criteria of the current meta-analysis affected the findings. Although the sample size for cases and controls in all eligible studies ranged from 18 to 243, the pooled ORs were not qualitatively altered by omitting the study of small sample. Three studies (Mabuchi et al., Al-Shahrani et al., and Dixit et al.) were not in HWE; however, the overall association was unchanged after the exclusion of these studies, which indicated that the results from this meta-analysis were statistically robust. Moreover, the heterogeneity test showed that there was no significant between-study heterogeneity in terms of the *MTHFR A1298C* polymorphism in the overall comparisons and subgroup analyses (**Table 3**).

**Publication Bias**

**Fig. 3:**
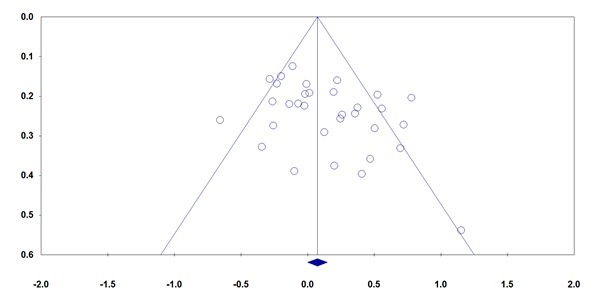
Begg’s funnel plots (publication bias) for the association of MTHFR C677T polymorphism with risk of glaucoma under allele model (T vs. C)AA)

We have used both Begg’s funnel plot and Egger’s test to access the small study effects of articles in literature. The shape of the fun¬nel plots did not reveal an obvious asymmetry. Then, the Egger’s test was used to provide statistical evidence of funnel plot symmetry. Egger’s test found evidence for the publication bias between MTHFR C677T polymorphism and glaucoma risk under the allele model (T vs. C: PBegg = 0.052, PEgger = 0.031, **Fig. 3**), homozygote model (TT vs. CC: PBegg = 0.010, PEgger = 0.008) and the recessive model (TT vs. CT + CC: PBegg = 0.022, PEgger = 0.005). This finding might be a limitation for this meta-analysis because studies with null findings, especially those with small sample size, are less likely to be published. The Duval and Tweedie non-parametric “trim and fill” method was used to adjust for publication bias. Meta-analysis with and without “trim and fill” did not draw a different conclusion, indicating that our results were statistically robust. Moreover, no significant publication bias for *MTHFR A1298C* polymorphism was found by Egger’s test in the overall or subgroup analyses.

## Discussion

To the best of our knowledge, this is the first and most comprehensive meta-analysis assessing the associations of *MTHFR C677T* and *A1298C* polymorphisms with risk of different types of glaucoma. A total of 33 case-control studies in 19 publications (3,504 cases and 2,525 controls) and nine case-control studies in six publications (1,073 cases and 775 controls) have investigated the associations of *MTHFR C677T* and *A1298C* polymorphisms with glaucoma risk, respectively. Our meta-analysis showed that *MTHFR C677T* polymorphism was not associated with glaucoma risk. Similar results were observed in the subgroup analyses based on ethnicity and types of glaucoma (POAG, PACG, PEXG, and NTG). However, we have found that the *MTHFR A1298C* may be associated with an increased glaucoma risk overall and by ethnicity. Moreover, in a subgroup analysis of glaucoma types, *MTHFR A1298C* polymorphism was significantly associated with an increased risk of POAG, but not with PXFG and NTG subgroups.

Interestingly, stratified analysis according to genotyping technique revealed a significantly increased risk of glaucoma in participants with the C677T polymorphism in those studies involving PCR-RFLP under recessive genetic model (TT vs. TC+CC: OR = 1.438, 95% CI 1.056-1.958, P = 0.021). With the recent advent of sophisticated high-throughput genotyping technologies such as semi nested PCR, the TaqMan allelic discrimination test, or real-time PCR, we may witness a significant progress in the association studies in the future [**32**]. High sensitivity of real-time PCR makes the technique applicable to very small samples [**33**]. However, this trend is possible because studies involving Caucasians mainly utilized Real-Time PCR. While, in studies involving Asians, PCR-RFLP was the main genotyping technique. We proposed that the sensitivity and specificity of genotyping techniques are further explored to seek out optimal approaches that could minimize the genotyping errors. Therefore, this result should be carefully interpreted and confirmed by conducting a further analysis of additional published studies. Moreover, the population based subgroup analysis also revealed that the presence of the *MTHFR C677T* was related to a higher risk of glaucoma under heterozygote genetic model (TC vs. CC: OR = 1.350, 95% CI = 1.012-1.802, P = 0.041). Similarly, Huo et al. suggested that there were significant associations between *MTHFR C677T* polymorphism and POAG in allelic genetic model and additive genetic model for population-based subgroup, which indicated that the T allele or TT genotype might increase the risk of POAG [**34**].

Pathogenesis of POAG is a complex process. It is known that genetic factors play an important role in POAG susceptibility [**35**]. However, most of the molecular mechanisms leading to POAG development are still unknown [**36**]. It seems that approximately 5% of POAG is currently attributed to a single-gene or Mendelian forms of glaucoma. Gene mutations in various loci have been identified by genetic studies and a genetic basis for glaucoma pathogenesis has been established [**18**,**37**]. Although many epidemiological studies have been conducted to assess the roles of *MTHFR C677T* polymorphism and POAG risk in different populations, results have been inconclusive. Recently, in a case-control study of 144 POAG cases and 280 controls in Saudi Arabia, Al-Sharani et al. indicated that the allele T and genotype CT of *MTHFR C677T* polymorphism confer risk of POAG, while allele C and CC genotype had a different role [**30**]. However, four studies did not find an association between *MTHFR C677T* polymorphism and POAG risk in Iranian, Mexican, Indian and Greek populations [**25**,**27**–**29**]. In 2012, Xu et al. have conducted the first meta-analysis including ten studies with 1,406 cases and 1,216 controls on *MTHFR C677T* polymorphism [**38**]. They found no impact of *MTHFR C677T* polymorphism on POAG susceptibility in the pooled analysis. Since then, a series of better-designed case-control studies on the association between *MTHFR C677T* polymorphism and POAG were performed. In the current meta-analysis, 16 eligible studies with 2,179 cases and 2,069 controls were identified and analyzed. The present meta-analysis suggested that there was no significant association between *MTHFR C677T* and POAG risk in the overall comparisons. Consistent with our study, a previous meta-analysis was undergone in 2015, which included 13 studies with 1,970 POAG patients and 1,712 control subjects, suggesting that the *MTHFR C677T* was not associated with increased genetic susceptibility to POAG [**39**]. However, we found out they wrongly included one study evaluated about the *MTHFR C677T* polymorphism and PACG risk in their meta-analysis. Our literature search was more thorough, containing four more articles, which increased the total number of cases and controls, thus, providing a greater power to our conclusions. Moreover, we used one more genetic model, the allele genetic model, to gain a more comprehensive and accurate understanding of the *MTHFR C677T* polymorphism association.

Assessing heterogeneity in the meta-analysis of genetic associations is critical for model selection and interpretation of the results. On the other hand, heterogeneity and publication bias might influence the results of the meta-analysis. It is well known that different factors, such as population stratification, source of controls, population size, deviation from Hardy–Weinberg equilibrium, and other covariates could be the source of heterogeneity. In the current meta-analysis, moderate between-study heterogeneity was detected across studies under allele, heterozygote and dominant genetic models for *MTHFR C677T* polymorphism and thus we selected the random-effects model to summarize the ORs. Therefore, we performed a meta-regression analysis to find the source of between-study heterogeneity. The results showed that ethnicity, glaucoma types, genotyping methods, source of controls and HWE status did not contribute to substantial between-study heterogeneity in the current meta-analysis.

It was obvious that some limitations of this meta-analysis should be considered. First, the sample size reported in literature is still relatively small and might not provide sufficient power to estimate the association between the null *MTHFR A1298C* polymorphism and the glaucoma risk. Second, the language of the publications was limited to English. Third, the current meta-analysis was based predominantly on Asian and Caucasian research. No study from other parts of the world was found, such as the Africans. This suggested a partial result that is only relevant to the Asian and Caucasian subgroups. Forth, the existence of between-study heterogeneity in some comparisons might compromise the reliability of conclusion. Finally, glaucoma is a multifactorial disease that results from complex interactions between various genetic and environmental factors. Due to the unavailability of other detailed information, our results were based on single-factor estimates without adjustments for other risk factors. Further evaluation of glaucoma risk should pay more attention to the potential interactions among gene–gene, gene–environment, and even different polymorphism of the MTHFR gene and other loci. Despite these limitations, our meta-analysis had some clear advantages. Our meta-analysis contained the largest sample size to date to assess the association between the *MTHFR C677T* and *A1298C* polymorphisms and glaucoma risk.

In summary, the current meta-analysis indicated that *MTHFR C677T* might not be associated with the glaucoma risk, and yet the *MTHFR A1298C* polymorphism may be a risk factor for glaucoma. In the future, large sample studies should be warranted to investigate the association of *MTHFR C677T* and *A1298C* polymorphisms with glaucoma, and to examine the potential gene-gene and gene-environment interactions.

**Funding**

No specific funding was obtained to support the conduct of this study.

**Conflict of interest**

The authors declared that there is no conflict of interest.
